# Use of a plastic scintillator detector for patient‐specific quality assurance of VMAT SRS

**DOI:** 10.1002/acm2.12705

**Published:** 2019-09-20

**Authors:** Jesse D. Snyder, Rodney J. Sullivan, Xingen Wu, Elizabeth L. Covington, Richard A. Popple

**Affiliations:** ^1^ Department of Radiation Oncology The University of Alabama at Birmingham Birmingham AL USA

**Keywords:** dosimetry, quality assurance, radiosurgery, scintillator, volumetric‐modulated arc therapy

## Abstract

**Purpose:**

To evaluate a scintillator detector for patient‐specific quality assurance of VMAT radiosurgery plans.

**Methods:**

The detector was comprised of a 1 mm diameter, 1 mm high scintillator coupled to an acrylic optical fiber. Sixty VMAT SRS plans for treatment of single targets having sizes ranging from 3 mm to 30.2 mm equivalent diameter (median 16.3 mm) were selected. The plans were delivered to a 20 cm × 20 cm x 15 cm water equivalent plastic phantom having either the scintillator detector or radiochromic film at the center. Calibration films were obtained for each measurement session. The films were scanned and converted to dose using a 3‐channel technique.

**Results:**

The mean difference between scintillator and film was ‒0.45% (95% confidence interval ‒0.1% to 0.8%). For target equivalent diameter smaller than the median, the mean difference was 1.1% (95% confidence interval 0.5% to 1.7%). For targets larger than the median, the mean difference was ‒0.2% (95% confidence interval ‒0.7% to 0.1%).

**Conclusions:**

The scintillator detector response is independent of target size for targets as small as 3 mm and is well‐suited for patient‐specific quality assurance of VMAT SRS plans. Further work is needed to evaluate the accuracy for VMAT plans that treat multiple targets using a single isocenter.

## 1 INTRODUCTION

Stereotactic radiosurgery (SRS) using a linear accelerator (linac) has a long history, beginning with the use of cones to collimate the beam. The development of narrow leaf width multileaf collimators (MLCs) and computer optimization enabled high‐quality patient‐specific plans, particularly for simultaneous treatment of multiple targets. When combined with volumetric‐modulated arc therapy (VMAT) and flattening filter free beams, these plans can be delivered very efficiently, on a timescale comparable to conventionally fractionated IMRT plans.^1^, ^2^

A significant challenge for quality assurance of SRS plans is the small target size. For techniques using a fixed collimator, such as a cone, the output factor must be accurately determined. For patient‐specific optimized plans, the current standard of care requires physical measurements to assess to the accuracy of dose delivery.^3^, ^4^, ^5^, ^6^ Detectors typically used for absolute dosimetry are not water equivalent and perturb the electron transport in small fields, resulting in a response dependent on the field size. In the well‐defined geometry used for output factor measurement, the field size dependence can be corrected using factors determined by Monte Carlo calculations.^7^ However, for patient‐specific quality assurance (QA), the geometry and field size are not sufficiently well‐defined to apply a correction factor. Radiochromic film (RCF) can be used for patient‐specific QA, but it requires careful calibration and is labor intensive.

Lack of water equivalence is the fundamental source of field size dependence. Miniature plastic scintillators have been developed that are nearly water equivalent.^8^, ^9^ A commercial version of the detector described by Beddar et al., having dimensions 1 mm diameter and 3 mm long, has been investigated for measurements of depth dose and profiles of small fields,^10^ determination of field size correction factors for other detector types,^11^ small‐field dose measurements in heterogeneous media,^12^ and for patient‐specific QA of small‐field SRS plans.^13^ Recently, a second generation has been developed that is smaller, 1 mm instead of 3 mm length. The near water equivalence coupled with small dimensions make this detector a promising candidate for point dose measurements of SRS plans, particularly those for which the dose distribution has significant dose gradient over the distance of 3 mm.

## 2 MATERIALS AND METHODS

**Figure 1:**
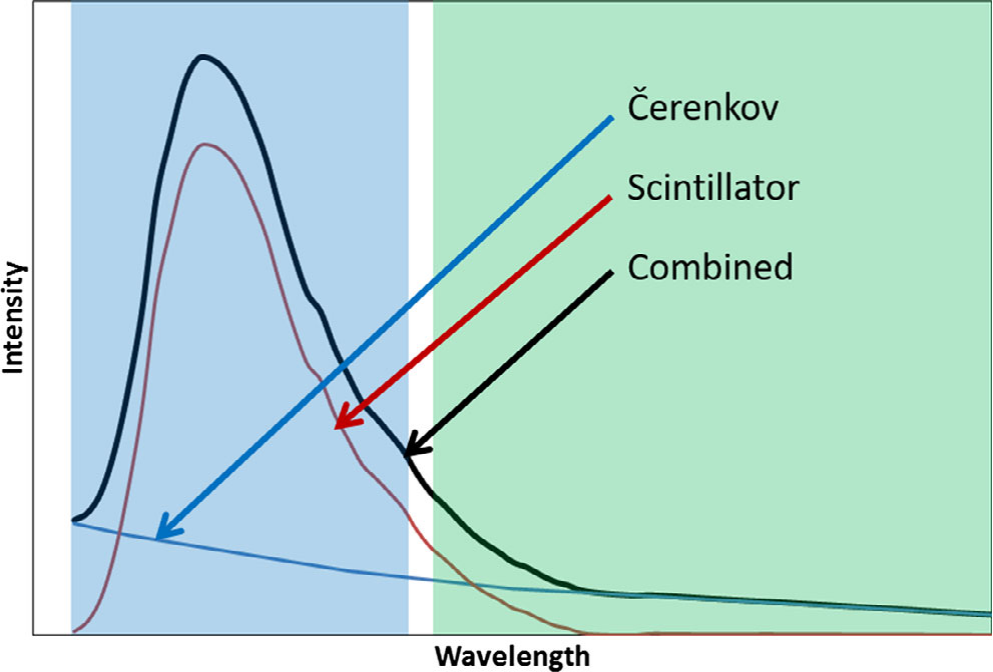
Schematic of the scintillator and Cerenkov radiation spectra along with the blue and green filter regions.

The active scintillator volume of the prototype detector (model W2, Standard Imaging, Madison, WI) was a cylinder of 1 mm diameter and 1 mm height. The scintillator was bonded to a 1 mm diameter polymethyl methacrylate (PMMA) optical fiber that was approximately 1 m long. The light output of the fiber was split between two photodetectors. One detector had an optical filter designed to transmit wavelengths in the range of the scintillation spectrum. This is referred to as the blue channel. The second detector collected the signal from the longer wavelengths, and is referred to as the green channel. The spectra of the scintillator and Cerenkov radiation are shown schematically in Fig. 1. The Cerenkov radiation has a broad spectrum that overlaps the scintillation spectrum and so the signal from the blue channel (*s*_Blue_) results from both scintillation and Cerenkov radiation, whereas Cerenkov radiation is the primary source of signal from the green channel (*s*_Green_). The portion of *s*_Blue_ due to Cerenkov radiation is proportional to *s*_Green_. The scintillator output is proportional to the dose deposited in the scintillator volume. The dose may therefore be determined from the signals by^14^, ^15^

(1) $$D=k_{\text{gain}}\left(s_{\text{Blue}}-k_{\text{CLR}}\cdot s_{\text{Green}}\right)$$

Rearranging,

(2) $$s_{\text{Blue}}=k_{\text{CLR}}\cdot s_{\text{Green}}+\frac{D}{k_{\text{gain}\;}}$$

The Cerenkov light radiation correction factor *k*_CLR_ can be determined by irradiating the detector using two field geometries that result in the same dose at the detector position but irradiate different lengths of the fiber. The correction factor is then given by

(3) $$k_{\text{CLR}}=\frac{s_{\text{Blue}}^{\max}-s_{\text{Blue}}^{\min}}{s_{\text{Green}}^{\max}-s_{\text{Green}}^{\min}}$$

where *max* and *min* refer to the signals for maximum and minimum volume of fiber irradiated, respectively. Once *k*_CLR_ is known, *k*_gain_ can be determined by irradiating the detector to a known dose:

(4) $$k_{\text{gain}}=\frac{D_{\text{ref}}}{\left(s_{\text{Blue}}^{\text{ref}}-k_{\text{CLR}}\cdot s_{\text{Green}}^{\text{ref}}\right)}$$

Alternatively, the Cerenkov light radiation correction factor *k*_CLR_ and signal to dose conversion factor *k*_gain_ can be determined by irradiating the detector to a known constant dose for range of field sizes and fitting a line to Eq. (2). Substituting into Eq. (1), the dose is given by

(5) $$D=\frac{\left(s_{\text{Blue}}-k_{\text{CLR}}\cdot s_{\text{Green}}\right)}{\left(s_{\text{Blue}}^{\text{ref}}-k_{\text{CLR}}\cdot s_{\text{Green}}^{\text{ref}}\right)}D_{\mathrm{ref}}$$

All measurements were done with the detector located at the center of a water equivalent plastic phantom having dimensions 20 cm × 20 cm × 15 cm high. For measurements in the vicinity of large dose gradients, typical of SRS, the detector must be accurately positioned with respect to isocenter. The detector was water equivalent and could not be visualized radiographically. Therefore, a dummy fiber provided by the manufacturer having a 1 mm diameter stainless steel ball located at the effective measurement point was used. Orthogonal kV images were acquired, the treatment table was shifted to place the ball at isocenter, and the dummy fiber replaced with the active detector. The accuracy of positioning using the dummy fiber was assessed by placing the detector in a 1.5 cm diameter cylindrical sleeve of water equivalent plastic that was mounted to a micrometer and obtaining readings in a 10 cm × 5 mm MLC defined field over a range of longitudinal positions.

For patient‐specific quality assurance of VMAT SRS, non‐coplanar arcs having table angles in the range [0, 90] and [270, 360) degrees are the norm. Hence, the detector can receive radiation from any direction within a 2π solid angle. The length of fiber irradiated increases significantly as the irradiation direction becomes parallel to the fiber. We investigated the effect of irradiation direction on determination of *k*_CLR_ using square fields 2 × 2, 4 × 4, 6 × 6, 8 × 8, and 10 × 10 cm^2^ with the central axis at 0°, 15°, 30°, 45°, 60°, 75°, and 90° to the fiber axis, where 90 degrees is perpendicular to the fiber.

Sixty VMAT SRS plans were selected from our clinical database. The plans were for treatment of single targets ranging from 3 to 30.2 mm equivalent diameter (median 16.3 mm), where the equivalent diameter is the diameter of a sphere having the same volume as the target. The prescription doses ranged from 5 to 20 Gy per fraction. All plans used two arcs, one of which was a full arc (spanning 358°) at table angle 0°. For one plan, the second arc was an additional full arc at table angle 0°. For the remaining plans, the second arc was a half arc (spanning 180°) having table angle at least 45° offset from 0. The most common angles were 70° (26 plans), 290° (12 plans), 300° (8 plans), and 80° (4 plans). The dose at isocenter was measured for each plan using the prototype detector. For each measurement session, the factor *k*_CLR_ was determined using Eq. (3) by irradiating the detector with a 2 cm × 7 cm field at collimator angles 0° and 90° and the dose conversion factor *k*_gain_ was determined using Eq. (4) by irradiating the detector to 5 Gy with a 4 cm × 4 cm field. The dose distribution of each plan was also measured in the coronal plane using radiochromic film (EBT‐XD, Ashland Chemical, Covington, KY). A calibration curve was obtained at each measurement session for each individual sheet of film. The film was scanned using an Epson model V700 PhotoPerfection document scanner (Epson America, Long Beach, CA) and converted to dose using a 3‐channel technique.^16^ To reduce the effect of pixel‐to‐pixel uncertainty and to approximate the measurement volume of the W2, the film dose was calculated using the average in a 1 mm × 1 mm region‐of‐interest (approximately 14 pixels) centered on isocenter. The dose distributions in the phantom were calculated using the Eclipse treatment planning system AAA algorithm version 13.6 (Varian Medical Systems, Palo Alto, CA). Because of the range of prescription doses, the measured dose values are reported relative to the calculated dose at isocenter. To assess the measurement uncertainty, the measurements were repeated on different days 10 times for each of four targets having equivalent diameters 3, 6, 18, and 29 mm,

## 3 RESULTS

**Figure 2:**
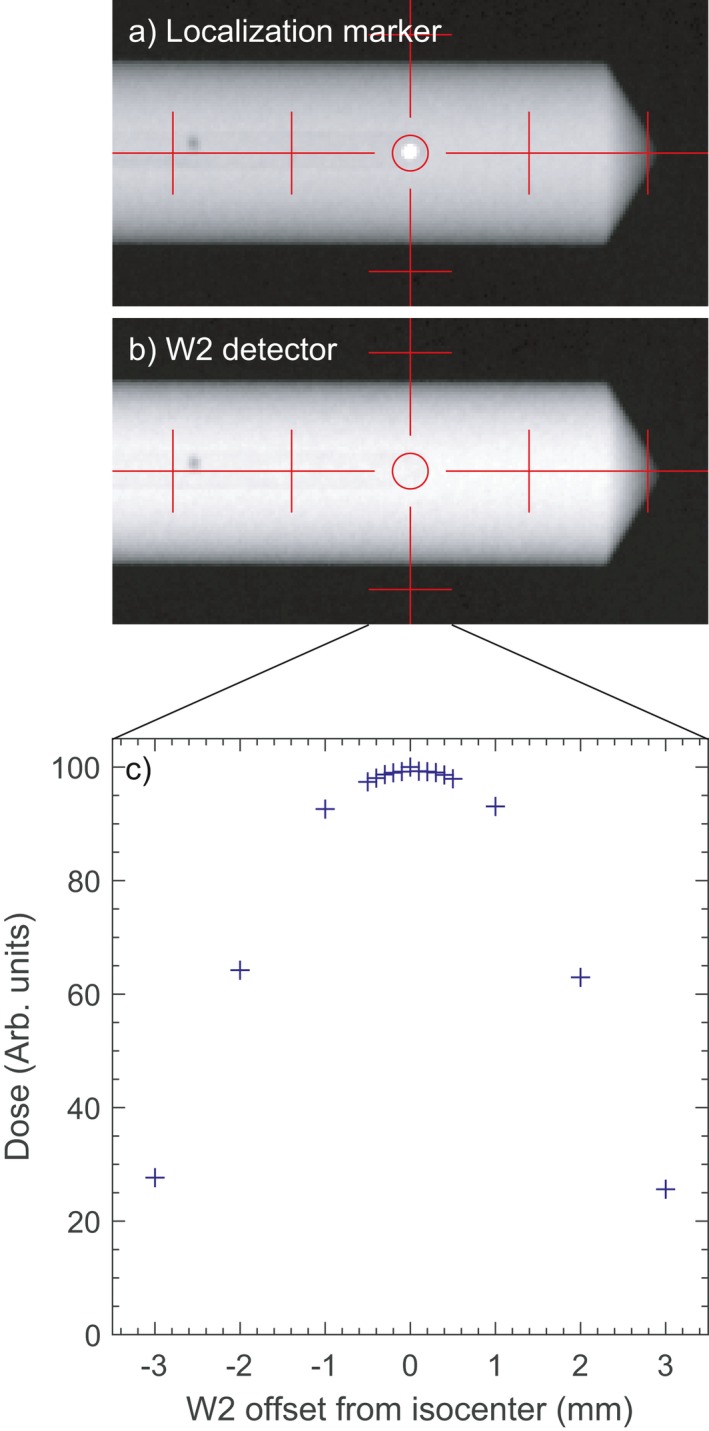
Images of (a) dummy detector with 1 mm stainless steel ball at detector position and (b) W2 detector, and (c) dose measured by the W2 as a function of detector offset from isocenter.

Images of the dummy detector with a 1 mm BB at the effective measurement point and of the actual detector are shown in Fig. 2, along with the measured dose as a function of position relative to the imaging isocenter. The BB position corresponded to the position of maximum dose to within 0.05 mm, demonstrating that the sensitive volume of the detector can be positioned with high accuracy using the dummy detector.

**Figure 3:**
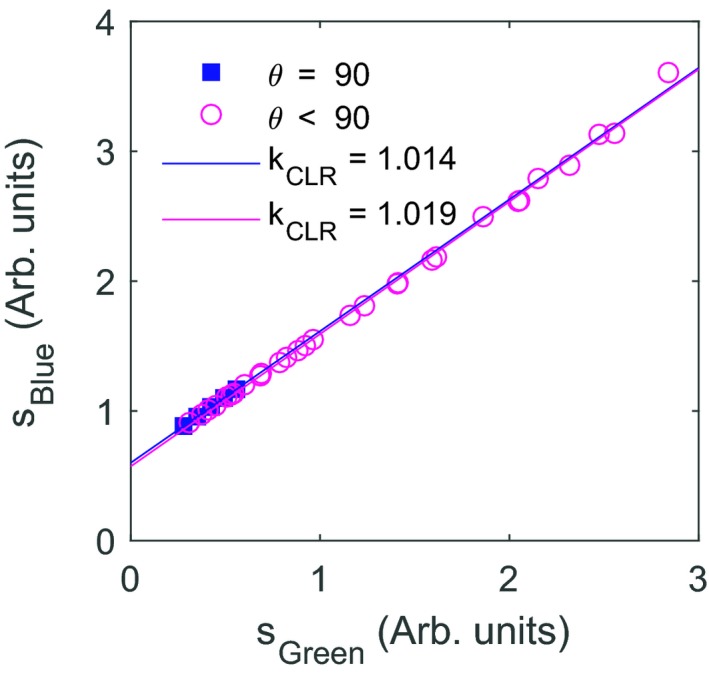
The blue channel signal as a function of the green channel signal for square fields 2 × 2, 4 × 4, 6 × 6, 8 × 8, and 10 × 10 cm^2^ with the central axis perpendicular (blue squares) and at 0°, 15°, 30°, 45°, 60°, and 75° (magenta circles) to the fiber axis, where 90° is perpendicular to the fiber, along with fits to Eq. (2) for the perpendicular fields (blue line) and the nonperpendicular fields (magenta line).

The blue and green channel signals are plotted in Fig. 3 for square fields having irradiation angles ranging between parallel and perpendicular to the fiber. A fit to Eq. (2) for all fields gives *k*_CLR_ = 1.015, whereas a fit limited to fields perpendicular to the fiber gives *k*_CLR_ = 1.014 and a fit for nonperpendicular fields gives *k*_CLR_ = 1.019.

**Figure 4:**
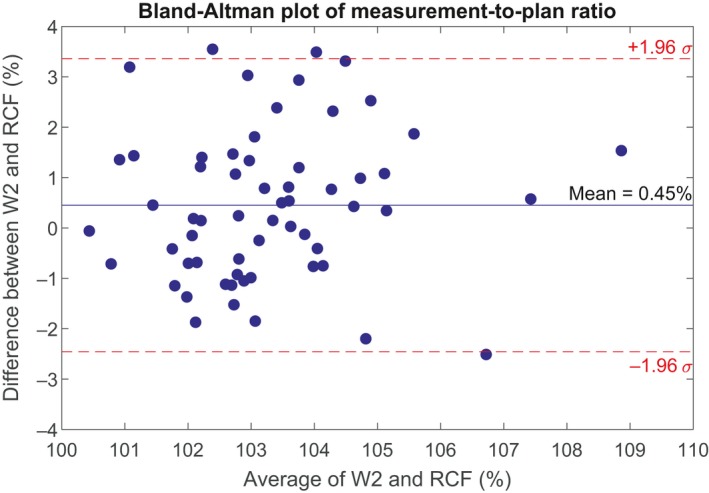
Bland–Altman plot of W2 and radiochromic film doses relative to calculated dose at isocenter.

**Figure 5:**
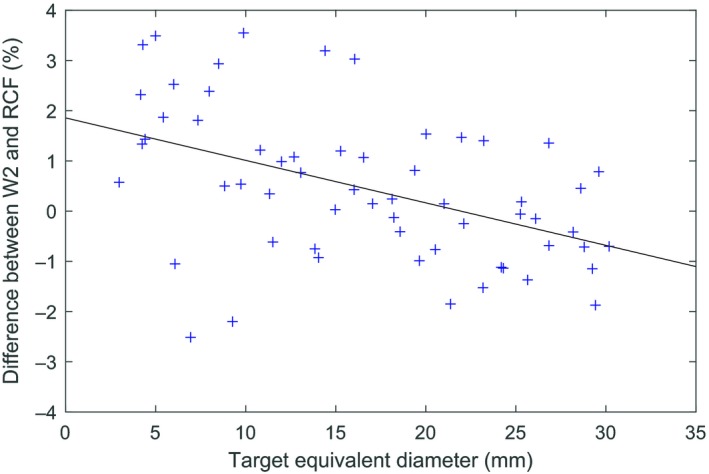
Difference between the W2 and radiochromic film dose relative to calculated dose at isocenter versus target equivalent diameter.

A Bland–Altman plot of the W2 and radiochromic film measurement‐to‐plan ratios for the 60 patient plans is shown in Fig. 4. The mean difference between W2 and film was 0.45%, with a 95% confidence interval −0.07% to 0.8%. For targets smaller than the median target size (16.3 mm), the mean difference of the ratios was 1.1%, with 95% confidence interval 0.5% to 1.7%. For targets larger than the median target size, the mean difference of the ratios was −0.2%, with 95% confidence interval −0.6% to 0.2%. A two‐sample *t*‐test indicates that the difference between the two groups is statistically significant (*P* < 0.001). The difference between the W2 and radiochromic film measurements as a function of target size is shown in Fig. 5. A linear fit to the data suggests a weak dependence on target size for which the difference decreases by 0.8% per cm increase in equivalent target diameter with Pearson correlation coefficient −0.47.

**Figure 6:**
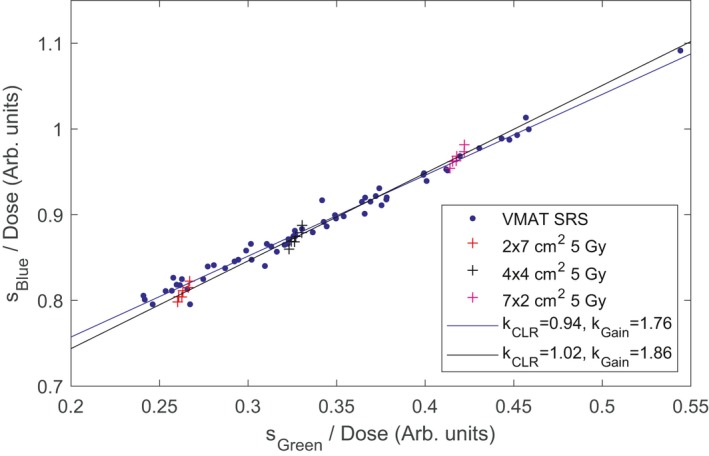
The blue channel signal as a function of the green channel signal for the 60 volumetric modulated arc therapy (VMAT) Stereotactic radiosurgery (SRS) plans (filled blue circles) and the calibration fields (crosses). The VMAT SRS plans are normalized to the measured radiochromic film dose and the calibration fields to the known dose. Also shown are fits to Eq. (2) for the VMAT SRS plans (blue line) and the calibration fields (black line).

**Figure 7:**
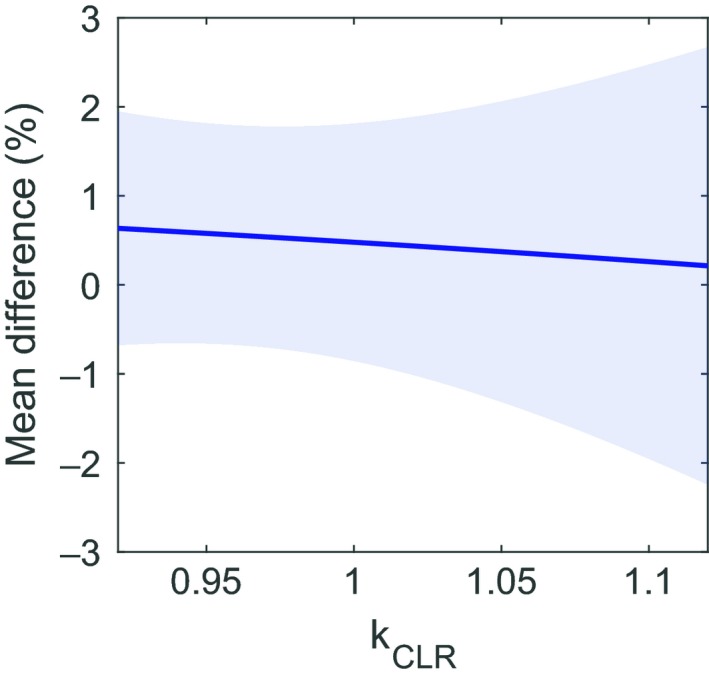
Mean difference between the W2 and radiochromic film as a function of fixed *k*_CLR_ and *k*_gain_ calculated using the session‐specific reference field. The shaded region shows the standard deviation.

The blue and green channel signals normalized to the film dose are shown in Fig. 6, along with the signals for the calibration fields used to obtain *k*_CLR_ and *k*_gain_. A fit to Eq. (2) gave *k*_CLR_ 0.94., whereas the mean *k*_CLR_ obtained from the calibration fields was 1.020, ranging from 1.013 to 1.025. To investigate the sensitivity to *k*_CLR_, the doses were recalculated using Eq. (5) for a range of values of *k*_CLR_. Note that Eq. (5) uses the session specific reference field, implicitly calculating *k*_gain_ for a specified *k*_CLR_. The change in the W2 dose relative to film is shown in Fig. 7. For *k*_CLR_ in the range from 0.96 to 1.02, the mean difference between W2 and film changed by 0.12%.

The repeated measurements are summarized in Table 1. It was noted that the dose distributions were not well centered at isocenter and that the measurement location could have a dose gradient. The magnitude of the dose gradient at the measurement location is also given in Table 1.

**Table 1:** Standard deviation of 10 repeated measurements and dose gradient at the measurement location.

Target equivalent diameter (mm)	Standard deviation of W2 measurement relative to mean	Dose gradient relative to dose at measurement location
3.0	1.3%	15.4%/mm
6.1	2.0%	18.4%/mm
18.1	0.3%	1.6%/mm
28.8	0.4%	2.5%/mm

## 4 DISCUSSION

Figures 3 and 6 show that the linear relationship between *s_Blue_* and *s_Green_* holds over a range of irradiation conditions. However, residual deviations from the linear fit suggest that the detector should be calibrated under conditions having a similar contribution to the signal by Cerenkov radiation as the plans to be measured. Figure 6 demonstrates that the VMAT SRS plans result in Cerenkov radiation contribution to the detector signal similar to that of uniform fields irradiating 1 to 3.5 cm of the fiber. The dose normalized signal for the 4 cm × 4 cm field falls approximately in the middle of the range, indicating that it is an appropriate choice for calibration.

The standard deviation of the repeated measurements given in Table 1 demonstrates that the uncertainty of the W2 < 0.5% in regions of low‐dose gradient. However, in high‐dose gradient locations, the uncertainty will be larger due to positioning uncertainty. In the work reported here, the detector was placed at isocenter, which was not always at the low gradient center of the dose distribution. Accuracy of the measurement would likely be improved if the measurement location was shifted to the position of the maximum dose. It is important to note that at this location, a detector positioning error will always result in underreporting of the dose.

The results reported here are consistent with those for the first generation plastic scintillator detector W1 (Standard Imaging, Madison, WI). The W1 is similar to the W2 but is 3 mm long. Qin et al found that the W1 agreed with RCF to within 3%, with an average difference of 0.31 ± 1.20%.^13^

The population of plans used in this study were designed for treatment of single targets at the isocenter. Plans that treat multiple targets using a single isocenter have larger irradiation volumes and spatially separated regions of high dose. These plans will generate different Cerenkov radiation conditions, particularly if a high‐dose volume overlaps the fiber but not the scintillator. Further work is necessary to evaluate the accuracy of the scintillator detector for measurement of multiple target, single isocenter VMAT SRS plans.

## 5 CONCLUSION

The scintillator detector is well‐suited for patient‐specific quality assurance of VMAT SRS plans. The detector response is nearly independent of target size for targets as small as 3 mm. Because the detector is near water equivalent, a dummy detector having a high‐density fiducial at the location of the scintillator is necessary to position the detector using a kilo‐voltage image guidance system. The signal due to Cerenkov radiation generated in the optical fiber is similar to that generated by uniform fields smaller than 7 cm × 7 cm. Further work is needed to evaluate the accuracy of the scintillator detector for multiple target, single isocenter SRS.

## CONFLICT OF INTEREST

The authors declare no conflict of interest.
